# World Market Development and Consumer Acceptance of Irradiation Technology

**DOI:** 10.3390/foods5040079

**Published:** 2016-11-24

**Authors:** Behnoush Maherani, Farah Hossain, Paula Criado, Yosra Ben-Fadhel, Stephane Salmieri, Monique Lacroix

**Affiliations:** INRS-Institut Armand-Frappier, Research Laboratories in Sciences Applied to Food, Canadian Irradiation Centre, 531 des Prairies, Blvd. Laval City, QC H7V 1B7, Canada; behnoush.maherani@iaf.inrs.ca (B.M.); farah.hossain@iaf.inrs.ca (F.H.); paula.criado@iaf.inrs.ca (P.C.); yosra.ben-Fadhel@iaf.inrs.ca (Y.B.-F.); stephane.salmieri@iaf.inrs.ca (S.S.)

**Keywords:** irradiated foods, food quality, nutritional value, bacterial radiosensitivity, consumer acceptance, consumer response, consumer communication, global perception

## Abstract

Food irradiation is an efficient technology that can be used to ensure food safety by eliminating insects and pathogens to prolong the shelf life. The process could be applied to fresh or frozen products without affecting the nutritional value. Presently more than 60 countries have adopted the technology. However, the technology adaptation differs from one country to another and, in some cases, consumers’ misunderstanding and lack of acceptance may hinder the technology adaptation process. This review summarizes the development of irradiation treatment worldwide and consumer attitudes towards the introduction of this technology. Also, the wholesomeness, beneficial effects, and regulation of irradiation are assessed.

## 1. Consumers’ Acceptance of Novel Agri-Food Technologies

In recent decades novel technologies have emerged worldwide in food production, processing, and preservation. These technical innovations are in development as a result of modern demands for foods that are fresher, have higher nutritional value, and are more natural with minimum food additives and no toxins or allergens [1,2]. As a result of these emerging technologies, higher-quality foods are produced with safer attributes since they have an extended shelf life and are sold at a reasonable cost. According to Rollin et al. [3], the use of novel foods or novel food ingredients in Europe and their marketing within the European Community was first defined by Regulation (EC) No. 258/97. In this legislation, novel foods or food ingredients are defined as those containing or produced from genetically modified organisms (GMOs), with a new modified primary molecular structure, consisting of or isolated from microorganisms, fungi or algae, plants, or animals not obtained by traditional propagation or breeding practices, and having a history of safe use. They also encompass processes that give rise to significant changes in composition or structure of a food or its ingredients. Research in this field is expanding, with food safety being a major driver for the development of new food technologies in order to reduce, control, and eliminate foodborne pathogens.

These novel technologies have the ability to enhance the nutritional values of food, lower the carbon footprint in food production, and reduce water consumption in food production lines. According to Agriculture and Agri-Food Canada [4], new processing and production technologies are being applied in the form of extraction methods and ingredient processing such as the development of water-soluble lipids for addition to foods and beverages, the application of pulsed ultraviolet light to improve the nutritional content of mushrooms, the conducting of genetic manipulation, stem cell applications, and cloning in breeding techniques, and the enhancement of delivery systems for bioactive ingredients through the use of nanoemulsions or microencapsulated probiotic cultures or organogels to preserve and extend food shelf life. Some of the novel food technologies highlighted by Frewer et al. [5] include genetic modification, animal cloning, nutrigenomics, nanotechnology, high-pressure processing (HPP), and pulsed electric field processing (PEF). These technical innovations are revolutionizing the food industry and impart a more competitive edge to agri-business.

The human diet has drastically changed over the years since the Industrial Revolution due to changes in agricultural production and animal breeding methods [6]. With the diversification of agricultural food practices and improvement in transport, farm products have since then moved easily between cities and across continental borders. Moreover, some means of conservation technologies such as refrigeration were welcomed with enthusiasm by consumers. However, an increasing body of literature suggests that consumers tend to oppose newly developed food technologies such as genetic modification of crops, which has stirred much controversy among consumers [6,7]. According to Slovic et al. [8], general consumers often evaluate the risks of novel technologies differently from experts. Erdem et al. [9] are also of the same view, and opined that the general public often diverges from experts in the evaluation of food processed by new technologies. Consumers are mostly of the view that risks stem from farming practices and processing, while farmers, on the other hand, believe that the greatest food safety risks occur as a result of consumer and processor actions. The lack of knowledge on the part of consumers and poor communication from the farmers and food processing technologists or engineers increases the misunderstanding between these two groups. To exemplify this aspect, a joint meeting in 1997 involving the World Health Organization (WHO), the Food and Agricultural Organization (FAO), and the International Atomic Energy Agency (IAEA) clearly determined that food irradiated with an appropriate dose to achieve the intended objective was both safe to consume and nutritionally adequate [10]. In fact, irradiation technology has proved to be efficient in reducing bacterial contamination to produce sterile food, which is particularly important for patients with impaired immunity such as those suffering from AIDS and cancer [3]. However, research has shown that the public tends to be averse to irradiated food despite the fact that it has been recognized as safe by authorities [11]. A number of factors may explain this reluctance, one of which is the lack of proper knowledge about the technology employed to process the food. According to Frewer et al. [5], public awareness is not high about food irradiation, and many people do not even know what irradiation is. A recent study by Nayga et al. [12] showed that awareness about the nature and benefits of food irradiation led to positive changes in consumers’ perception and influenced their decisions to buy irradiated food.

Novel agri-food technologies has led to the development of food preferences and food neophilia (individuals being willing to try new foods) versus neophobia (individuals being reluctant to try novel foods) [13]. Based on these concepts, Pliner and Hobden [14] even introduced a scale to measure food neophobia. Cox and Evans [15] modified this approach to establish a measure of food technology neophobia. This scale gauges the fear and reluctance of consumers to eat foods produced by innovative technologies. In a study conducted by Capiola and Raudenbush [16], it was found that food neophilics and food neophobics tend to exhibit different sensory evaluations, psychophysical ratings, stimulus sampling, physiological responses, and genetic predispositions.

## 2. Factors Driving Consumer Response to Emerging Technologies

For years investigators have conducted research in view of assessing consumer responses to novel food technologies, and examined a variety of factors that may influence the perception of consumers towards these emerging technologies. In this context, the risks associated with these responses have been evaluated based on different scenarios such as voluntary and involuntary, immediate or delayed, observable or unseen, fatal or non-fatal, the degree to which the risk is known or not, and the degree of control that the consumers have over the risk [11,17]. A range of foods processed by different technologies such as irradiated food, genetically modified food, food treated by pulsed electric fields and ultraviolet laser, and microbially contaminated foods have been under scrutiny to assess their perceived risks and consumers’ concerns [18,19,20]. Based on a study conducted by Cardello et al. [21], 20 different traditional and novel food processing technologies were evaluated for their acceptance by consumers. Genetic food manipulation was at the top of the list and elicited the highest level of concern from consumers, followed by the addition of bacteriocins, irradiation, and pulsed X-rays. Consumers displayed less concern about technologies such as UV light, pulsed electric fields, and oscillating magnetic fields. The perception of food technologies by consumers in turn plays a crucial role in their choices, purchasing behavior, and acceptance of these foods. From an economic point of view, it is essential to optimize the sensory quality of food products so as to foster their consumption by the public. According to Bruhn [22], good flavor or unique flavor combinations of food products largely determine their success with consumers. Such intrinsic features also include the nutritional value of the food products such as fiber, beneficial fatty acids, lycopene, vitamin C, and probiotic, among others [23]. An increasing number of consumers are opting for more “natural” food products with no food additives or those that have been produced in environmentally friendly or sustainable ways. In addition, food convenience also plays an important role, such that over 80% of consumers, in a study, indicated that their purchase was heavily dependent on convenience [24].

However, relying only on the sensorial quality of foods does not guarantee their success in the marketplace since there are other factors that contribute to their acceptance by consumers. According to Cardello [21], contextual, cognitive, social, cultural, and attitudinal attributes also drive consumers’ food choices. With regards to novel food technologies, consumers show concern about the nature of the resulting processed food or the nature of the processing technology itself, and these play a crucial role in determining whether consumers will buy the food or not. Studies have shown that a lack of knowledge among consumers regarding novel food processing technologies is a major impediment to their acceptance [25]. Hence, consumer communication is essential for consumers to accept innovative food technologies. To some extent, the dread/control framework may explain the aversion of consumers to new food technologies. Many consumers have little knowledge about modern production agriculture; according to Campbell and Fitzgerald [26], the new technologies applied in food processing are foreign to contemporary consumers, and the low literacy of consumers often limits their acceptance.

According to Li-Cohen and Bruhn [27], information about the processing of food should be presented to consumers through different routes depending on age and gender. Based on this study, it was found that men and younger consumers tend to prefer web-based sources than women or middle to older people, who rely more on television, newspapers, magazines, and supermarket brochures. In addition, consumers appear to be cautious about accepting novel technologies applied to food based on the perceived risks and lack of benefits. A study conducted by Cox et al. [28] showed that males, on average, display less concern about these emerging technologies but give more importance to the cost and size of the food products. On the other hand, females show more negative beliefs about innovative food technologies.

## 3. Overcoming Aversion of Novel Foods: Communication

In an effort to mitigate the negative perception of the impact of foods derived from novel technologies and inculcate trust among consumers, food industry researchers are focusing their attention on public education and dissemination of information regarding these food products [29,30,31]. Educational programs should be set up to impart the right information to the public regarding food produced from novel technologies. Very often, the future benefits of the technology are praised rather than the immediate, direct consumer benefits. According to Bruhn [22], emphasis should be laid on immediate consumer benefits, and the information should be imparted to them using layman-suitable terminology. In addition, to maintain the trust of the public, it is imperative to be transparent and share both what is known and what is not known with regard to the risks and benefits. The food industry should also anticipate and respond in a timely manner to concerns about potential risks expressed by the public. According to Costa-Font et al. [32], proper labeling can effectively provide information about the technology employed and its benefits to raise awareness and enhance transparency. Curtis et al. [33] and Frewer et al. [34] prefer food products to display clear and detailed labels. Consumers tend to accept the associated risks if they are aware of them and have control of them. Studies have shown that if these approaches are implemented, the aversion of consumers towards novel products decreases and their acceptance increases in general [35,36]. Educational and other information-based approaches to changing consumer attitudes appear to work in most cases. However, studies performed by Grunert et al. [19] and Wilson et al. [37] on consumer responses to genetic modification of food have shown that addressing the information deficit does not completely reassure consumers. Nonetheless, it has not been shown that the same situation applies to other emerging food processing technologies such as food irradiation. In the latter case, Nayga et al. [12] showed that educating consumers about the nature and benefits of food irradiation may effectively induce a positive response and improve its acceptance.

## 4. Food Irradiation as a Safe Technology

Food irradiation is a processing technique that involves exposing food to ionizing radiation such as electron beams, X-rays, or gamma radiation to induce the demise of bacteria that can cause food poisoning, control insect infestation, delay fruit ripening, or prevent vegetables from sprouting [38,39]. Studies have shown that this technology can prevent the proliferation of microorganisms that cause food spoilage, such as bacteria and molds, by changing their molecular structure [40]. Also commonly known as “cold pasteurization,” it offers a wide range of benefits to the food industry and the consumer by ensuring the hygienic quality of solid or semi-solid foods through inactivation of foodborne pathogens [41]. Interest in irradiation food technologies is increasing because of persistently high food losses from infestation, contamination, and spoilage by bacteria and fungi, rising concern about foodborne diseases, and a growing international trade in food products that must meet strict import standards of quality and quarantine. In all these areas, food irradiation has demonstrated valuable and practical benefits when integrated within an established system for the safe handling and distribution of food products [42]. In addition, with increasingly restrictive regulations or complete prohibition on the use of a number of chemical fumigants for insect and microbial control in the food industry, irradiation is becoming a preferred alternative to protecting food against insect damage and as a quarantine treatment for fresh produce [43,44]. As such, irradiation can help to ensure a safer and more plentiful food supply by extending food shelf life through the control of pests and pathogens. Importantly, according to the World Health Organization (WHO) and Food and Agriculture Organization (FAO) [10,39], it is a safe technology for the processing of food commodities when the appropriate radiation dose is respected.

## 5. Wholesomeness of Irradiated Food

Many studies have been conducted over the last 100 years on the ‘wholesomeness of irradiated foods’, a terminology developed during those efforts [45]. As mentioned before, irradiation is a non-thermal process utilized to achieve the preservation of food. Irradiation (even for radurization at 0.4–10 kGy and radicidation at 40–45 kGy) does not impart heat to the food and the nutritional quality of the food is generally unaffected [46]. The irradiation process can reduce microbial contamination in food, resulting in improved microbial safety as well as extended shelf life of the food. There is an established framework of international standards for food irradiation covering human health, plant protection, labeling, dose delivery, quality assurance, and facility management. Approximately 60 countries permit irradiation of one or more foods or food classes [47].

The Codex Alimentarius Commission (Codex) is the body responsible for standards related to human health. Food irradiation must be conducted according to good management practice and comply with the Codex Alimentarius General Principles of Food Hygiene [48]. The foundation for food irradiation was set with the adoption of the Codex World-wide General Standard for Irradiated Foods in 1983 [49] and a significant revision in 2003 [50]. The General Standard states that the minimum absorbed dose should be sufficient to achieve the technological purpose and the maximum absorbed dose should be less than that which would compromise consumer safety of wholesomeness or would adversely affect the structural and functional properties or nutritional and sensory attributes [50]. In 1983, the Codex Alimentarius Commission accepted that foods irradiated up to 10 kGy were safe and therefore toxicological testing was no longer necessary. In 1997, the United Nations confirmed that foods could be treated at any dose without any detrimental effect on the food’s wholesomeness. The study group concluded that high-dose irradiation, conducted in accordance with good manufacturing and irradiation practices, could be applied to several types of foods to improve their hygienic quality, make them shelf stable, and produce special products [51] (Table 1).

The FDA considers four broad areas to establish the safety of irradiated foods: radiological safety, toxicological safety, microbiological safety, and nutritional adequacy. No evidence of toxicity and radioactivity attributable to irradiation of food was found. Furthermore, under realistic conditions, radiation has been approved to achieve the intended microbiological effect by eliminating the *Clostridium botulinum* and its toxin as the most resistant bacterium and will not increase the microbiological risk [52,53,54].

### 5.1. Nutritional Aspects

Food irradiation is a technology that addresses both food quality and safety because of its ability to: inactivate the parasites, spoilage, and food-borne pathogenic microorganisms; and, under certain conditions, deactivate viruses, delay the ripening of fruits, inhibit germination (e.g., onion, garlic), and control the post-harvest losses caused by insect infestation without significantly affecting the sensory or other organoleptic attributes of food, thus contributing to improvements in food hygiene and enhancing public health [40,55,56]. Furthermore, irradiation treatment can stimulate the biosynthesis of bioactive compounds [38]. In some conditions, irradiation can also activate the synthesis of phenolic compounds and enhance the vitamin content in fruits and vegetables [38]. Recent studies showed that radiation treatment generally increased the levels of certain beneficial phytochemicals and enhanced the biological properties of some plants with nutritional value [57]. Indeed, the addition of any energy to food can break down its nutrients. Foods are irradiated to provide the same benefits (such as destroying pathogenic bacteria) as when they are processed by other technologies such as heat, refrigeration, freezing, or chemical treatment. Indeed, non-thermal food treatments have no potentially harmful residues and, also in these techniques, nutrient losses are relatively small and often substantially less than the nutrient losses associated with other methods of preservation, such as canning, drying, and heat pasteurization and sterilization [56,58]. Furthermore, it should be noted that the main advantage of food irradiation is that it can be used to treat packaged foods, which will remain safe and protected from microbial contamination after treatment [59]. From a nutritional point of view, trace elements and minerals are not affected by irradiation. Macronutrients such as protein, carbohydrates, and fats are not significantly affected by doses up to 50 kGy [52,55]. Saturated and mono-saturated fatty acids represent the essential content of neutral lipids in meat. Different studies on meat irradiation and its effect on lipids have shown that at low radiation doses, lipids in the presence of their natural protectors are not particularly sensitive to radiation-induced peroxidation. They also found no significant difference in total saturated and unsaturated fatty acids between irradiated (1, 3, or 6 kGy) and un-irradiated frozen chicken muscle [60,61]. Proteins are built of amino acids, which are the essential nutrients for the body. The effect of radiation on protein is related to their state, structure, and composition, whether native or denatured, whether dry or in solution, whether liquid or frozen, and to the presence or absence of other substances. However, long-term feeding studies also concluded that irradiation of raw and prepared meat, including precooked shrimp and chicken, to prolong shelf life, does not lead to a reduction in their protein nutritional value and no distinct decrease of the biological value of proteins was observed [62,63,64]. The amount of vitamin loss due to food irradiation is affected by several factors, including doses, temperature, presence of oxygen, and food type. Generally, radiation at low temperatures in the absence of oxygen reduces any vitamin loss in foods, and the storage of irradiated foods in sealed packages at low temperatures also helps prevent future vitamin loss. However, not all vitamins have the same sensitivity to irradiation [65] (Table 2).

Most of the studies confirmed that irradiated foods are generally nutritionally equivalent or even better than non-irradiated foods that are subjected to normal processing [57,64,66,67]. Finally, according to a collective agreement between the Food and Agriculture Organization (FAO) of the United Nations, the International Atomic Energy Agency (IAEA), and the World Health Organization (WHO), on the basis of knowledge derived from over 50 years of research, irradiated foods were considered safe and wholesome at the specified radiation dose [68]. A joint FAO/IAEA/WHO Study Group on High-Dose Irradiation (JSGHDI) stated that any food treated at any high dose is acceptable and healthy as long as it is palatable. This statement acknowledges that any food destroyed by inappropriate irradiation treatment may have lost its essential properties but is not necessarily hazardous for consumption [45,69].

### 5.2. Sensory Aspects

The consumer attitude towards food is very complex as it is influenced by sensory and non-sensory attributes, as well as by the interactions between them. Recently, many studies on sensory acceptance of radiated foods and the influence of food irradiation on consumer behavior have been performed [70]. The findings confirmed that food irradiation is a technology that addresses both food quality and safety because of its ability to control spoilage and food-borne pathogenic microorganisms without significantly affecting the sensory attributes or other organoleptic attributes of the food [56,71]. Many studies found no significant difference in sensory quality and protein content of stir fry chicken dices and ground meat after irradiation during the storage time [71,72].

The irradiation of vegetables, nuts, green tea, grains, and fresh and dried fruits (such as spinach leaves, carrots, lettuce, broccoli, red kidney beans, raisins, pistachios, dried figs, apricots, apples, and pears, and fresh strawberries, pineapples, clementines, and mangoes) was also shown to lead to good sensory and organoleptic quality acceptance [40,66,67,70,73,74]. Furthermore, in some cases, radiation processing also leads to an increase in the nutritional values of irradiated fruits and vegetables, such as vitamin C content and phenolic compounds [38,75,76].

### 5.3. New Perspectives

Irradiation combined with other processes can contribute to food safety, improve the nutritional value of products, and control losses during transportation and commercialization [77,78,79]. Many studies have demonstrated that depending on various added compounds such as essential oils (EOs) extracted from plants and the combined treatment used (e.g., modified atmosphere packaging (MAP), mild heat treatment), the relative bacterial radiosensitivity (RBR) increased 2 to 4-fold [79,80]. The findings showed that the combined treatment leads to a decrease in the needed dose of radiation, obviates the need for high-heat treatment, and finally protects the nutritional values and sensory quality of natural products, thereby obtaining higher quality products. Recent studies have approved radiation in combination with mild heating treatment or the addition of some EOs such as carvacrol or cinnamon to increase bacterial radiosensitivity (RBR) [79,80].

Many studies have shown that irradiation technology in combination with other treatments such as mild heat treatment can be used as an innovative and effective method to reduce or eliminate the growth of bacteria and parasites and subsequently extend the shelf life of food products with acceptable nutritional values [81].

## 6. Food Irradiation around the World

According to the Institute of Food Science & Technology (IFST) [82], more than 50 countries have given approval for over than 60 products to be irradiated in the world. In Asia the use of irradiation for food decontamination and phytosanitary purposes was estimated to 285,223 tons per year in 2010. In the European Union, the quantity of irradiated foods was estimated to 9264 tons, especially for spice decontamination. In the USA the total was estimated at 103 tons [83]. The USA, China, The Netherlands, Belgium, Brazil, Thailand, and Australia are the major countries that have adopted the technology commercially [82]. The use of irradiation for phytosanitary purposes is important around the world. More than 18,446 tons of food are irradiated worldwide for phytosanitary purposes, representing 5734 tons in Hawaii, 493 tons in Australia, 100 tons in India, 951 tons in Thailand, 850 tons in Vietnam, and 10,318 tons in Mexico, mostly for export to the USA [83]. Australia was the first user of irradiation for phytosanitary purposes in 2004, especially to export to New Zealand. India started to export to the USA in 2007, followed by Thailand and Vietnam. Mexico started to ship irradiated foods to the USA in 2008 and the export increased from 257 tons in 2008 to 3521 tons in 2009, making it now the most important exporter to the USA.

### 6.1. Food Irradiation in America

According to Kume et al. [83], the USA has an important commercial irradiation program and information is distributed via an update newsletter. Around 120,000 tons of food are irradiated annually in the USA for human and animal consumption [84]. The most important irradiated products in the USA are spices (80,000 tons), pet treats (20,000 tons), fresh products (14,000 tons), and ground beef (8000 tons). Approval for food irradiation started in the USA in 1963 for wheat and wheat flour at a dose of 0.5 kGy, and in 1985 for parasite elimination at 1 kGy. Then, from 1985 to 1992, the irradiation of dry enzymes, fresh products, spices, and poultry at doses of 10, 1, 30, and 3 kGy, respectively, was accepted. From 2000 to 2008 red meat, eggs, seeds for sprouting, pet food, sweet potatoes, shellfish, lettuce, and spinach were added in the list of approved irradiated foods at doses of 8, 50, 1, 5.5, 4, and 1 kGy, respectively. In 2012, the Food and Drug Association of the USA extended its approval to cover irradiated unrefrigerated meat. However, according to IFST [82], an outbreak of *E. coli* O157:H7 in 1993 that resulted in four deaths and hundreds of hospitalizations, attributed to undercooked hamburgers, was a major stimulus to adopt the irradiation of foods. Following a 2006 outbreak of spinach contaminated with *E. coli*, approval of the irradiation of spinach and lettuce was given in the USA in 2008. In 2009, approval of irradiation of oysters to eliminate *Vibrio vulnificus* was given. Presently, more than 6000 tons of products including papaya, sweet potatoes, basil, ginger, melons, taro leaves, curry leaves, longan, litchi, mangosteen, and rambutan are irradiated annually in Hawaii [85]. The irradiated products are exported to the U.S. mainland, Germany, and Switzerland.

In Canada around 2000 tons of spices are irradiated annually. Only potatoes, onions, wheat, flour, flour, whole and ground spices, and dehydrated seasoning preparation are approved for irradiation. The regulations were adopted in 1960 and 1965 to treat potatoes and onions, respectively, at a dose of 0.15 kGy; in 1969 to treat wheat and flour at a dose of 0.75 kGy; and in 1984 to treat spices and seasoning at a dose of 10 kGy. A regulatory proposal was submitted in 2002 for ground beef, poultry, shrimp, prawns, and mangoes but is still under consideration.

In Mexico, around 7500 tons of irradiated guavas, mangoes, and peppers are irradiated for export to the USA [82]. According to Kume and Todoriki [83], exports increased to more than 10,318 tons, especially guava (9121 tons), sweet lime (600 tons), mangoes (239 tons), grapefruit (101 tons), and manzano peppers (257 tons).

In Brazil, quantity of irradiated foods was estimated at 20,000 tons for spices, 3000 tons for fruits, and 23,000 tons in total in 2009 [86].

The total quantity of irradiated foods in the USA, Canada, and Brazil in 2009 was around 101,400 tons of spices, 7000 tons of fruits, and 8000 tons of meat, for a total of 116,400 tons of food [86]. According to this research, as spices are mainly used in industrial processed foods, special labeling is not required for them; however, irradiated fruits and meat should be labeled.

### 6.2. Food Irradiation in Asia

More than 285,223 tons of foods were irradiated in Asia in 2010 [83]. According to this research, China is the largest Asian producer of irradiated foods, with more than 100 irradiators to irradiate more than 200,000 tons of garlic, spices, dried vegetables, cooked meats, fruits, and grain [83]. Vietnam also irradiated more than 66,000 tons of foods, including frozen seafood and fruit in 2010. Japan irradiated around 6246 tons of potatoes. In Indonesia similar amounts of food were irradiated in 2010, especially cocoa, frozen seafood, spices, and others. Then, India and Thailand irradiated around 2000 tons of spices, dried vegetables, fruits (Mango, Mangosteen, Logan), fermented sausage (Nham), herbs, sweet tamarind, and others in 2010. Pakistan, Malaysia, and the Philippines irradiated around 1000 tons of food, including spices, fruits, nutritional drinks, herbs, and dried vegetables [83]. The authorization can, however, include more items. For example, in Pakistan the regulations include potatoes, onions, fresh fruits, grains, chicken and meat, fish and seafood, spices, herbs, and dry food for animals [87]. In Korea, 5394 tons of spices and dry vegetables were irradiated in 2009 [86]. However, the authorized products in Korea include potatoes, onions, garlic, chestnuts, fresh and dried mushrooms, dried meats, powdered fish, shellfish, soybean paste powder, hot pepper powder, soybean sauce powder, starch, dried spices and vegetables, yeast and enzymes, powdered aloe, ginseng, and sterile meals for hospital patients. The irradiation of these products was accepted from 1987 to 1995 [87]. Bangladesh obtained authorization for food irradiation in 1995. In 1998, around 1300 tons of different foods including frozen foods were irradiated [87].

### 6.3. Food Irradiation in Australia

From 2004 to 2010, 256–1205 tons of mangoes, litchi, and papaya were imported to New Zealand. In 2010, the irradiation of mango and litchi was 460 tons and 33 tons, respectively [83]. In 2012, the Food Standard Authority in Australia and New Zealand also approved the use of irradiation for capsicum and tomatoes [88].

### 6.4. Food Irradiation in Africa and Other Regions

Eighteen thousand tons of spices and honey were irradiated in 2009 in South Africa. Egypt also uses the technology for the irradiation of spices and dehydrated vegetables (550 tons/year). In the Ukraine, more than 70,000 tons of grain and fruits are treated by irradiation annually [86].

### 6.5. Food Irradiation in the European Union

Around 9264 tons of food were irradiated in the European Union in 2010 [89]. Ten countries are doing commercial application of food irradiation. The most important countries are Belgium, France, and The Netherlands. In Belgium, irradiation treatment is especially performed for frog legs, poultry, herbs and spices, dehydrated blood, fish and shellfish, and meat (7279 tons/year). In The Netherlands, irradiation is practiced for dehydrated vegetables, frog parts, spices, herbs, egg white, poultry, and shrimp (3299 tons/year). In France, frozen frog legs, poultry, Arabic gum, herbs, spices, and dried vegetables are treated by irradiation (3111 tons/year). Spain, Poland, Hungary, Germany, the Czech Republic, Romania, and Estonia especially treat herbs, spices, and vegetable seasoning (about 369, 687, 151, 127, 27, 10, and 17 tons/year, respectively) [83,86].

## 7. Global Perceptions of Food Irradiation

Food irradiation has been approved since 1989 by the USDA and FDA. Irradiation treatment is widely applied for blood and spices. However, this technology is still controversial due to its bad reputation such as modification of food properties, formation of dangerous substances, and fall out dangerous process or accidents and its association with the nuclear establishment. Actually, new research has demonstrated that almost all of these prejudices are misleading statements and overestimated. However, recent studies have shown that consumers still remain reluctant to purchase irradiated products. This is intimately related to the lack of information about irradiation process and the natural human resistance to change. In fact, the perception of irradiated food by consumers depends on the degree of awareness of people about irradiation technology. However, due to the growing number of recalls after food poisoning incidents, it is important to revise the marketing policy of irradiated food to make consumers more conscious of the benefits of this technology for human wellbeing. Based on a broad search, Heddle et al. [90] proposed six recommendations to increase the acceptance of irradiated food by consumers

1 Set up a public education campaign to address needs.
2 Develop a knowledge translation strategy for health care professionals.
3 Develop risk communication strategies to address risk perceptions.
4 Identify a strategy and focus of ongoing research and surveillance related to pathogen reduction.
5 Explore society’s willingness to pay attention to pathogen-reduction technology, by considering the economic impacts associated with this technology, including direct and indirect costs and the potential for offsetting additional costs by eliminating redundancy.
6 Consider the issue of choice.

Most studies on consumers’ perception about irradiated food have shown that education seems to be the key to consumer acceptance. Numerous consumer studies clearly showed that when given a choice and even a small amount of accurate information, consumers are not only willing to buy irradiated foods but also often prefer them over food treated by conventional means. A variety of market research studies conducted over the past four decades demonstrated that the majority of consumers will choose irradiated products over non-irradiated ones after they learn the facts and understand the benefits [91].

### 7.1. America

#### 7.1.1. South America

Thirty years ago, a study demonstrated that Argentinian people were impressed by irradiated onions and garlic. Six tons of onions and one ton of garlic were sold between 1985 and 1986 and global consumer appreciation gave interesting results. Recently, the work of Finten et al. [74] confirmed that, in Argentina, people have little awareness about this technology. Approximately 39% of respondents believed misleading myths about food irradiation and had doubts about it. However, after being supplied with informative materials, 42% of respondents were willing to purchase or eat irradiated ready to eat (RTE) spinach leaves, while 35% were doubtful. This emphasizes the importance of having well-informed and more aware consumers.

#### 7.1.2. North America

In the USA in the 1980s, irradiated apples sold well even at a higher price than unirradiated apples ($0.1/lb) [92]. This demonstrated that consumers are looking for a product of good quality and irradiation treatment is not a real barrier.

The approval of fresh meat and meat products irradiation in February 2000 allowed the control of meat pathogens. The challenge was that only half of adult resident were willing to buy irradiated ground beef or chicken and only a quarter were willing to pay extra for these products [93]. These statistics were discouraging for companies.

On the other hand, consumers have recently become more concerned about irradiation risks. According to Crowley et al. [94], acceptance of meat irradiation was clearly driven by concerns about the risks of irradiation, but not the risks of bacterial contamination, confirming differences in the perception of natural and technological risks. Thus, a general lack of concern about foodborne illness and the fear of perceived, possibly negative “radiation” side-effects impede willingness to endorse this food processing technology. The respondents are afraid of different aspects of the technological risks associated with irradiation such as over-irradiated meat, health risks associated with eating irradiated meat, radiation exposure due to accidents, and the belief that the negative health consequences of meat irradiation would be worse than its potential benefits.

Efforts are needed to educate people to improve their perception about irradiated foods. Thomson et al. [95] demonstrated that educators’ beliefs about the safety of food irradiation were influenced by their perceived understanding of it. In the USA, after a relatively short explanation about irradiation and alternative processes, consumers generally become more accepting of irradiation especially when compared to treatments that involve exposure of food to chemical additives and residues. The marketing of irradiated hamburger, Hawaiian papaya, and sweet potato was a great success for at least 10 years. New irradiated exotic fruits from Mexico and several Asian countries are now available in markets [69]. In Michigan and Florida, public education efforts have achieved some success in changing peoples’ attitudes about purchasing irradiated foods. One store’s efforts have enabled it to sell a variety of irradiated produce including grapefruits, oranges, onions, tomatoes, mushrooms, and blackberries [38]. The report of Hunter [96] showed that consumers’ most important motivations when buying irradiated food are killing foodborne pathogens (77%), controlling insect infestation (64%), and reducing the use of insecticides (60%). A study performed by the National Cattlemen’s Beef Association in 2002 reported that 85% of participants would accept irradiated beef if some improvements were made: 1—replacement of the word “irradiation”; 2—explaining the irradiation process; 3—giving consumers a choice between irradiated and non-irradiated beef; and 4—improvement of the quality of the final product [97].

Also, Canadian consumers are not really informed, as 57% of Canadians had not previously heard about irradiation. However, Canadian consumers of different genders and ages also appear to behave differently about accepting novel technologies, as men and people aged over 55 years old (48%) were more awarded. After a brief presentation about irradiation techniques, 74% of people aged over 55 years were ready to support food irradiation. In total, 66% of respondents supported food irradiation, against 34% who opposed this option [98].

### 7.2. Europe

Despite the effectiveness of irradiation for food decontamination, the limited diffusion between EU member countries of ionizing radiations for the treatment of agri-food reduces its popularity.

Italian people, for example, have a historical fear of nuclear technology, and too often consumers are misinformed about food irradiation technology. However, contrary to what one might imagine, in 1976 irradiated potatoes were appreciated by the Italian people because of their better quality and storability.

Irradiated foods have sold well in Poland (irradiated potatoes and onions in 1987–1988), with 90% overall acceptance for potatoes and 95% for onions; in France, irradiated strawberries sold well in 1987 even an additional 30% added to the conventional price, which indicates a good acceptance (only 2% of people rejected strawberry irradiation) [92]. Irradiated frog’s legs were also successfully retailed in France and Belgium.

In the United Kingdom, no significant difference was observed between chilled ready meals irradiated at 2 kGy and non-irradiated meals (carrots, broccoli, beef and gravy, roast potatoes, and Yorkshire pudding) [99]. Irradiation was found to be the least acceptable intervention by Scottish people [100].

Turkish people also have a lack of information about the irradiation process (only 29% of consumers are aware of food irradiation) [101]. In an evaluation, between 69% and 80% of respondents were very concerned and uncertain about the safety of irradiated foods and were cautious about what they purchase [101,102]. Only 11% expressed that irradiated foods are safe.

According to Parlato et al. [103], the anti-irradiation message can be effectively counteracted and consumer confidence in the safety of irradiation process can be restored by detailed science-based information on irradiation. This starts with a huge effort from the health authorities and other institutions to allow correct understanding of the potential advantages of irradiation for the necessary investment to occur.

### 7.3. Africa

Few studies have been carried out on the African continent. Between 1978 and 1988, 90% of South African consumers of irradiated potatoes, mangoes, papaya, and strawberries judged that they were satisfied with the quality of the irradiated items [92].

### 7.4. Asia

In Thailand, irradiated onions were well accepted by consumers in 1987 and respondents were ready to purchase them even at a slightly higher price than unirradiated onions [92]. Fermented sausage was also well accepted and over 94% of consumers indicated a willingness to buy irradiated sausage again.

In Bangladesh, irradiated fish was well accepted by consumers, especially for its better quality and appearance [92]. Also, when onions were sold, consumers were in favor of the irradiated onions. In China, spicy chicken feet were also well accepted by consumers. In the Philippines, for similar prices, irradiated onions and garlic were preferred to unirradiated ones.

In Korea, studies on irradiation acceptability to women demonstrated that it is more convincing to hear a lecture by an expert followed by watching a video- and reading a book with a group. In addition, acceptance of irradiated food has been shown to lead to support for the nuclear industry [104].

Japan invested in public education about radiation for schoolchildren and their parents. More than 60% of kids were satisfied with the information given. Consumers’ perceptions about irradiation thereby seemed to shift to become more positive [105,106].

### 7.5. Oceania

In New Zealand, irradiated mango and litchi have been imported and sold since 2005. Presently a significant volume of irradiated mangoes, lychees, tomatoes, and capsicums are now purchased by consumers in New Zealand. It is clear that irradiation fulfills not only a technological need but also a consumer need by making quality produce available at competitive prices. Consequently, a significant proportion of the New Zealand public will consistently buy irradiated fresh products when they are made available [107].

## 8. Communication: An Important Factor in Consumer Acceptance of Irradiated Food

In the last decade, with the awareness of health problems caused by food spoilage, the food industry has utilized new technologies to improve the safety and shelf life of food. Some of the new technologies being applied are biotechnology, ionizing radiation, pulsed electronic fields, ultraviolet laser treatment, etc. However, those emerging technologies pose challenges to the industry in terms of consumer choice and acceptability. One of the major barriers of commercialization is the influence on sensorial characteristics that influence the purchase of the product, but there are also some extrinsic factors that might influence consumers, including contextual, social, cultural, and attitudinal variables [21].

To evaluate the reaction of people, studies have evaluated the perceived risk to consumers of new food technologies [108,109]. Data showed that consumers have significant levels of concern about the hazards of new technology, which from a technical/rational point of view was evaluated as a low risk. In general, consumers perceived gamma irradiation as a risky technology because of (1) the carcinogenic nature of irradiated food; (2) the risks of using irradiation technology; (3) the risk of irradiation escaping; and (4) the risks associated with the transportation of radioactive material [110]. This negative perception of new technology was studied by Cardello [21], who related the perceived risks to the consumer’s lack of awareness about any processes applied to the purchased food—either because these are out of the consumer’s control or because they are unobservable. Therefore, consumers do not know if what they are consuming is irradiated or not or if it might have negative effects on their health.

Several authors [101,111,112] studied people’s opinions and readiness to accept irradiated products. Junqueira-Gonçalves et al. [113] indicated that the people surveyed had a lack of information and understanding of food irradiation. Briefly, 45.9% of the responders answered that irradiated food means radioactive food, and 57.1% of people were uncertain if gamma irradiation can cause damage to human health and/or the environment. Nevertheless, marketing has shown that consumers are willing to purchase irradiated products if they are informed about the effects and the process [91].

Several studies have demonstrated that food irradiation can mitigate the development of food-borne diseases, which continue to grow with approximately 76 million illnesses, 325,000 hospitalizations, and 5000 deaths in the United States annually [114], and 1.6 million illnesses, 4000 hospitalizations, and 105 deaths in Canada [115]. Even though scientists recognize food irradiation as a safe and effective process, there can still be either a negative bias or a lack of information, which can be a limiting factor when addressing consumer resistance towards food irradiation.

Scientists have realized that consumer perception of novel technologies relies heavily on the communication approach employed. It was proven by Furuta et al. [106] that more than half of interviewed people present at a “Radiation fair” held in Japan recognized the word “radiation,” which was taught to them in elementary school and by the mass media. In addition, results showed that image can be improved if correct information about radiation is relayed to the public. Hence, factors such as teaching, labeling, and food retail could play an important role in diffusing new technology and the creation of a positive link with consumers.

### 8.1. Teaching

Although gamma irradiation has shown many beneficial effects such as insect disinfection, extension of food shelf life, and reduction of bacteria [69], irradiation has not been widely adopted as a commercial process. It was suggested by Zimmerman et al. [116] that in terms of new food technology either cognitive or affective perceptions can have an influence on consumer attitudes. Similar findings were mentioned by Edwards [117], who demonstrated that people’s perceptions are usually based on the global attitude towards new technology when knowledge of the specific topic is low [118,119].

However, not only the global adoption of the novelty but also educational programs can have significant impact on consumer attitudes. Recent studies point out that once consumers are educated, they will buy irradiated food and adopt a positive attitude towards food irradiation [120,121]. With the aim of accomplishing this goal, scientists must take into account the non-technical perceptions of people about the believed “risk” of the gamma irradiation technique [122]. In comparison to other food procedures, gamma irradiation was ranked alongside food preservatives and sodium nitrate as a less-feared factor.

Although nowadays consumers are looking for food safety and “freshness,” there is evidence that consumers regard food safety as a basic requirement. Thus, consumers do not value the extension of product shelf life that results from gamma irradiation. In 1988, the United Kingdom was confronted with *Salmonella* infection in egg production, while consumers were not aware that this crisis could occur in eggs. This is the reason why highlighting improvements caused by irradiation in food safety can serve as a proof of the advantages of this technology.

Some authors such as Rogers [123] have proposed that the topic of gamma irradiation should be included in educational programs so as to introduce consumers to it. Rogers [123] mentioned the importance of including characteristics such as advantages, compatibility, complexity, trialability, and observability in the diffusion and adaptation of new technology. Complexity is defined as the degree to which an innovation is perceived as difficult to understand and use. Observability is defined as the degree to which the applied technology is visible to others; this will allow consumers to observe the process and the results of the irradiated food. Trialability is expressed by Rogers [123] as the degree to which the innovated product can be experienced on a limited basis. For instance, when a consumer has his first contact with irradiated food, his organoleptic senses will allow him to compare and determine his opinion with respect to irradiated and non-irradiated foods.

### 8.2. Labeling; RADURA Symbol

**Figure 1:**
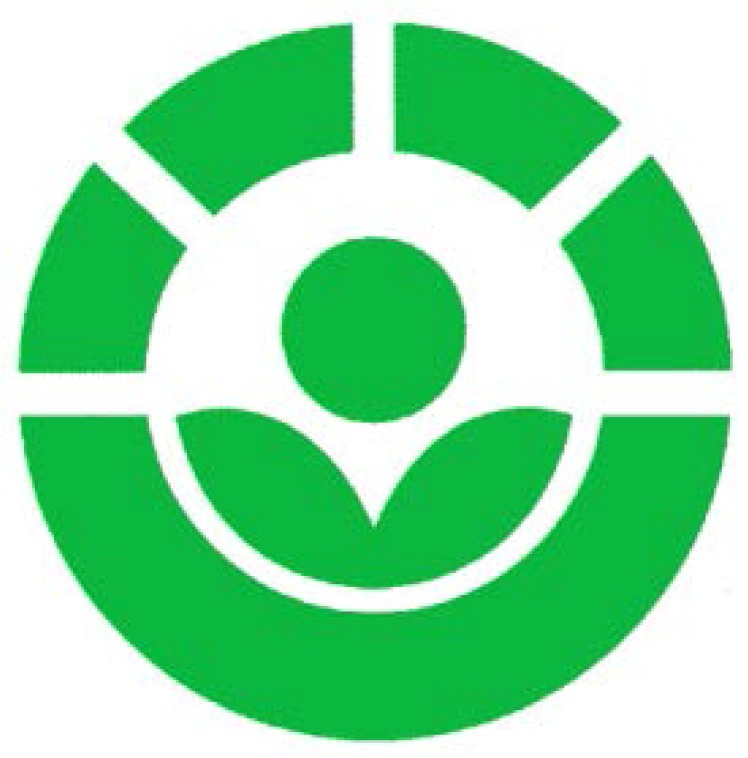
The RADURA symbol.

In order to identify an irradiated product and alert consumers to its quality, the Pilot Plant for Food Irradiation at Wageningen, The Netherlands, created the symbol RADURA (Figure 1). This word is related to “radurization,” a word derived from radiation and the Latin word “*durus*” for “lasting.” The term is used for the process of exposing food to ionizing radiation to enhance and extend the shelf life. Thus, the product is irradiated at doses in the range from 0.4 to 1 kGy to decreased number of spoilage bacteria [124]. Due to the fact that external microorganisms can also encounter the irradiated product, food packaging is part of the process. The “RADURA” symbol was established to represent the irradiation treatment. The symbol presents a plant (dot and two leaves), in a closed package (circle), irradiated with ionizing rays passing through the package to the food (dashed lines). Despite the fact that the use of the RADURA symbol is optional, according to the Codex Alimentarius standard [125], if the food or an ingredient product is treated with ionizing radiation a written statement shall be placed in proximity to the food to indicate that the treatment was done. This last requirement might change from country to country. For instance, in the United States, labeling is only required if the whole food has been irradiated and labeling is not required at restaurants/catering establishments [126]. Canada requires labels and written statements such as “irradiated,” “treated with radiation,” or “treated by irradiation” when the whole product was irradiated or more than 10% of the ingredients that compose the final product [127]. On the other hand, New Zealand demands labeling for even minor ingredients and in restaurant/catering establishments [32].

Labeling is an important step that assures consumers whether they are deciding to buy or not buy irradiated products. Indeed, once consumers know about the identification of products, it is easy for them to accept the risks of purchasing food derived from a new technology [128].

According to Junqueira-Gonçalves et al. [113] the RADURA symbol is not frequently present on food labels in Chile. However, studies mentioned that 55.8% of people would buy irradiated food because the symbol transmits the sensation of confidence and safety. Similar discussions were held by Rollin et al. [3], who said that labeling products treated with new technology will raise awareness and improve transparency. However, there are still ambiguous reactions demonstrated in the study performed by He et al. [128], who reported that over 30% of people in the USA would consider the term “irradiated” beef product as a warning and only 21% would consider it safe and buy it.

## 9. Food Retailers

Since food irradiation is not a familiar technology for everybody and does not show any detectable changes in food, consumers’ confidence relies on food processors who might inform them whether the product was irradiated or not [110]. Thus, the role of food retailers is as important as that of educators, giving a sense of assurance of food quality to people who still are scared and avoid the technology [129].

However, even if trust between food retailers and consumers is gained, it can be easily lost with a single mistake [129]. Trust-destroying mistakes such as withholding of information or scientific tests indicating that a product is less safe than originally conceived might affect consumer perceptions about gamma irradiation.

Thus, in order to be transparent and maintain consumers’ confidence, food retailers should firstly give consumers the freedom to choose between irradiated and non-irradiated products. For this, positive disclosure is required. Secondly, because irradiation is a process that does not exhibit any modification on food, retailers must indicate that the irradiation process has been done in an appropriate manner.

Moreover, food retailers are the first facilitators in developing irradiation acceptability by merchandising irradiated goods to the consumer. As the first contact of consumers with such product is in the market, food retailers should give consumers the opportunity to observe and judge for themselves about the new technology. Thus, consumer confidence in the food supply chain will be enhanced.

## 10. Future Directions for Gamma Irradiation

To encourage consumers to accept gamma irradiation, some authors have considered future strategies for increasing retail of irradiated food [69,110,130]:

- Highlight the advantages of the technology rather than pointing out the technology. Consumers value “freshness” more than increased shelf life, which can be seen as “unnatural.”
- Take into account the positive and negative aspects that will coexist in any food debate.
- Use labels to show advantage information, thus offsetting the warnings that labels are perceived to bring with them. A labeled product will be assured. , thus decreasing consumer opposition to irradiated food. This fact was observed in Australia and New Zealand.
- Create a partnership with food retailers so they can promote the marketing of irradiated food, especially to those who are small or medium-sized.
- It can be worthwhile to have stakeholders that believe in the value of food irradiation, thus food retailers will be seen as less biased and consumer trust will increase.

## 11. Conclusions

Foods processed by novel and emerging technologies, e.g., biotechnology, ionizing radiation, pulsed electric field, ultra violet laser treatment, etc. pose a serious challenge to factors such as consumer choice, purchasing behavior, and acceptance of irradiated foods. Future research on novel food processing and preservation technologies should focus more directly on questions and issues related to consumers’ expectations. Developing a better understanding of the variables that directly influence the acceptance of these products and more effective marketing and informational strategies could improve the acceptance of these novel technologies in tomorrow’s marketplace.

Evidence has shown that gamma irradiation, especially related to food, represents a hazardous technology to consumers. This is the reason why commercialization has encountered several barriers to being adopted and accepted by consumers. It has been demonstrated that communication, labels, and education about new technology will enhance consumers’ perception of irradiated food. Thus, showing the possible problems carried by food-borne diseases can create consciousness of the importance of food safety. On the other hand, due to the fact that gamma irradiation is a process that does not affect physical aspects of the product, the role of the food industry and food retailers should be to inform the public either by labeling products or by telling consumers about the benefits of the irradiation.

New strategies based on positive messages of gamma irradiation marketplace will thus encourage consumers to be more receptive to safety-enhanced and high-quality irradiated foods.

Based on knowledge derived from over half a century of research, irradiated foods are safe and wholesome at a specified radiation dose. The irradiated foods are generally nutritionally equivalent to non-irradiated foods subjected to normal processing, even better in some cases; radiation processing can lead to an increment in the nutritional value of irradiated fruits and vegetables such as vitamin C content and phenolic compounds.

Furthermore, many studies have shown that irradiation technology in combination with other treatments can be used as an innovative and effective method to add value to food products. As previously detailed, people are still confused and fail to differentiate irradiated foods from radioactive foods. When well informed, a reduced number of consumers will reject irradiated food. What a consumer is looking for is a product with good quality and a competitive price. When consumers are aware of the short- and long-term dangers of chemical additives, they accept more irradiation treatments being applied to food products. However, companies should update their quality system and implement new procedures to support risk management and the supply and distribution chains.

## Figures and Tables

**Table 1:** Foods permitted to be irradiated under FDA regulations (21 CFR 179.26). Data were updated by Komolprasert [46].

Food	Purpose	Dose
Fresh, non-heated processed pork	Control of *Trichinella spiralis*	0.3 kGy min. to 1 kGy max.
Fresh foods	Growth and maturation inhibition	1 kGy max.
Foods	Arthropod disinfection	1 kGy max.
Dry or dehydrated enzyme preparations	Microbial disinfection	10 kGy max.
Dry or dehydrated spices/seasonings	Microbial disinfection	30 kGy max.
Fresh or frozen, uncooked poultry products	Pathogen control	3 kGy max.
Frozen packaged meats (solely NASA)	Sterilization	44 kGy min.
Refrigerated, uncooked meat products	Pathogen control	4.5 kGy max.
Frozen uncooked meat products	Pathogen control	7 kGy max.
Fresh shell eggs	Control of *Salmonella*	3.0 kGy max.
Seeds for sprouting	Control of microbial pathogens	8.0 kGy max.
Fresh or frozen molluscan shellfish ^1^	Control of *Vibrio* species and other foodborne pathogens	5.5 kGy max.

^1^ Data provided by FDA [51].

**Table 2:** Relative sensitivity of vitamins to irradiation.

High Sensitivity	Low Sensitivity
Vitamin C *	Carotene
Vitamin B1 (thiamin) *	Vitamin D
Vitamin E	Vitamin K
Vitamin A	Vitamin B6 (pyridoxine) *
	Vitamin B2 (riboflavin) *
	Vitamin B12 (cobolamin) *
	Vitamin B3 (niacin) *
	Vitamin B9 (folate) *
	Pantothenic acid *

* Water-soluble vitamins, Fat-soluble vitamin. Updated from Woodside 2015 [55].
